# Emerging Microfluidic Plasma Separation Technologies for Point-of-Care Diagnostics: Moving Beyond Conventional Centrifugation

**DOI:** 10.3390/bios16010014

**Published:** 2025-12-23

**Authors:** Ergun Alperay Tarim, Michael G. Mauk, Mohamed El-Tholoth

**Affiliations:** 1 Department of Mechanical Engineering and Applied Mechanics, University of Pennsylvania, Philadelphia, PA 19104, USA; alperay@seas.upenn.edu (E.A.T.); mmauk@seas.upenn.edu (M.G.M.); 2 Health Sciences Division, Higher Colleges of Technology, Al Ain Zakhir Campus, Abu Dhabi 17155, United Arab Emirates; 3 Department of Virology, Faculty of Veterinary Medicine, Mansoura University, Mansoura 35516, Egypt

**Keywords:** plasma separation, microfluidics, diagnostic, lab-on-a-chip devices, point-of-care testing (POCT), personalized medicine

## Abstract

Plasma separation is an essential step in blood-based diagnostics. While traditional centrifugation is effective, it is costly and usually restricted to centralized laboratories because it requires relatively expensive equipment, a supply of consumables, and trained personnel. In an effort to alleviate these shortcomings, microfluidic and point-of-care devices offering rapid and low-cost plasma separation from small sample volumes, such as finger-stick samples, are quickly emerging as an alternative. Such microscale plasma separation systems enable reduced costs, rapid test results, self-testing, and broader accessibility, particularly in resource-limited or remote settings and facilitate the integration of separation, fluid handling, and downstream analysis into portable, automated lab-on-a-chip platforms. This review highlights advances in microfluidic systems and lab-on-a-chip devices for plasma separation categorized in design strategies, separation principles and characteristics, application purposes, and future directions for the decentralization of healthcare and personalized medicine.

## 1. Introduction

Plasma is the cell-free fraction of blood and serves as the gold standard for in vitro medical diagnostics due to its informative array of detectable biomarkers. The separation of blood plasma is an essential component of diagnostics and research in medicine as it allows for the differentiation of analytes, such as biomarkers, enzymes, and hormones, which are needed to diagnose diseases and metabolic states. Separated plasma is commonly used in tests for infections (e.g., HIV, hepatitis, and respiratory pathogens), therapeutic management, coagulation studies, genetic studies, nutritional studies, preventive medicine, and transfusion-related purposes [1].

The US FDA [2] recommendations for in vitro diagnostic devices emphasize the need for a simple operation that does not require sample manipulation, and should avoid the need for operator intervention, such as a separate centrifugation step to extract plasma from whole blood samples. This implies that diagnostic devices should use unprocessed raw samples, e.g., capillary blood draws, or alternatively, the integration of a plasma extraction process into the device for a fully automated, self-contained test. Accordingly, the development of a minimally instrumented plasma extraction process compatible with a microfluidic format would gain the advantage of using plasma for analysis, without sacrificing the anticipated portability, simple operation, rapid test results, and compact device features of a point-of-care device suitable for self-use by patients. The advantages of integrated plasma extraction processing include larger sample volumes (thus increasing sensitivity), removal of whole blood components such as heme that inhibit enzymes employed for nucleic acid amplification tests (NAAT), and better analysis reproducibility. A case in point is HIV viral load tests that require determination of virus in plasma rather than proviral DNA contained in white blood cells. Particularly, plasma extraction avoids issues with clogging microfluidic channels by cells and cell debris, removes components that may contribute spurious background fluorescence, optical scattering or absorption and interfere with optical detection, and allow for more precise sample metering for quantitative testing [1,3].

Plasma separation is an important process for increasing sensitivity and specificity of blood-based assays. By removing blood cells and cellular debris, plasma provides a clearer medium for analysis and increased accuracy of test results. Even for assays involving blood cell-related components, the use of plasma avoids interference in detecting extracellular markers. In addition, because most analytes of interest are uniformly distributed in plasma, smaller microliter volumes may provide adequate test sensitivity and reproducibility. Particularly, this enables the use of relatively small volumes of finger-prick-derived plasma (instead of venipuncture samples) for diagnostic screening applications, therapeutic monitoring, or research [4,5].

Blood sampling methods depend on the use and volume required. Traditional methods such as venipuncture and arterial sampling yield larger volumes, but are invasive, require trained people to use them, and generally are not appropriate for rapid on-site testing. Finger-prick sampling is a less invasive alternative that uses small blood volumes, is typically less painful for patients, and is well suited for microfluidics [5]. This is particularly relevant in point-of-care (POC) and self-testing situations where money, accessibility, and speed are priorities.

Most of the previous review articles in this field have approached microfluidic plasma separation from relatively specific or fragmented perspectives. For example, some reviews have focused on individual separation techniques [6,7,8]. Other reviews have provided overviews of earlier-generation techniques, which laid important foundations for the field but do not fully capture the most recent technological developments or current point-of-care design considerations [9,10]. Accordingly, a comprehensive literature synthesis that integrates both passive and active plasma separation methods, emphasizing modern and emerging microfluidic technologies rather than traditional centrifugation, is crucial for guiding current research and future device development in blood–plasma separation.

**Figure 1:**
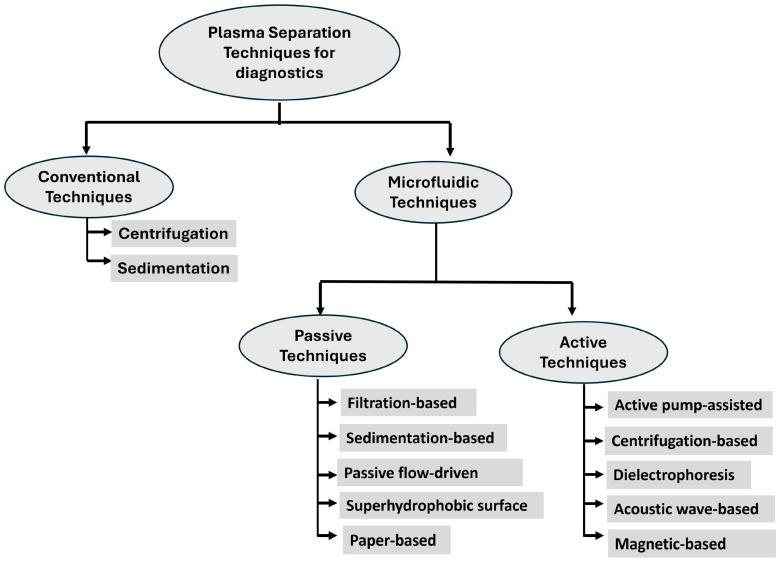
Overview of plasma separation techniques used in diagnostic applications.

In this review, we highlight novel approaches to plasma separation methods as they are incorporated into lab-on-a-chip platforms and microfluidic systems (Figure 1) designed for minimal blood volumes, and categorize these innovations based on their underlying principles and design strategies to support recent POC devices and miniaturized instruments. The purpose of this review is to provide a broad survey of this rapidly evolving field as it relates to POC microfluidic technology, and such that active and passive plasma separation methods can improve healthcare by providing access to diagnostics, improving earlier disease detection, blood related disease monitoring, and facilitating personalized medicine.

## 2. Conventional Techniques for Plasma Separation

**Figure 2:**
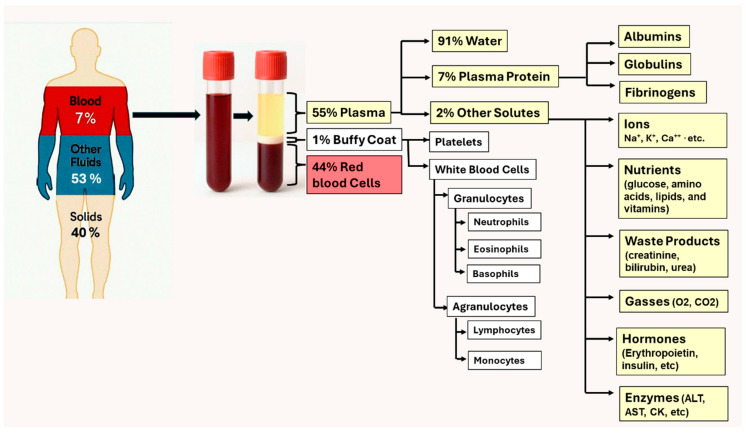
The fundamental composition of whole blood, including plasma and cellular elements.

Centrifugation and sedimentation are two widely used traditional methods for separating plasma. Following the principle of centrifugation, whole blood is processed in a centrifuge, and parts of the blood are isolated based on differential densities of blood components. Plasma is the supernatant (Figure 2), and the bottom layer is leukocytes and erythrocytes [11]. There are different types of centrifuges for plasma separation depending on the application. The most common are bench-top clinical centrifuges, which are standard for blood analyzing laboratories. Refrigerated centrifuges are used for the plasma portion which contains temperature-sensitive components, such as proteins and nucleic acids. Other centrifuges, such as high-speed and microcentrifuges, manage smaller volumes more efficiently. More recent developments focus on portable centrifuges for smaller, low-resource settings [12]. Alternatively, sedimentation depends on the autonomous setting of cellular parts into the bottom layer of a column, with the aid of gravity, and can use supplementing techniques such as density gradients with differing densities [7,13]. However, the use of these conventional techniques is largely restricted to laboratories for primary plasma separation due to their relatively high equipment costs, long processing times, and the need for trained personnel to safely handle the equipment [8]. Consequently, various techniques based on microfluidic or paper-based devices have been developed to address the needs of POCT.

## 3. Microfluidic Techniques for Plasma Separation

Plasma separation within microfluidic devices can be accomplished through a variety of techniques. Microfluidic platforms offer powerful tools, leveraging both inherent fluid phenomena (surface tension, capillarity, laminar flow) to achieve effective separation at small scales. These methods are explored, first covering those that rely on passive fluid manipulation, such as size-based and flow-driven passive filtration, gravitational sedimentation, and combinations thereof. Subsequently, we examine those incorporating active components, including actuated flow manipulation, centrifugation, dielectrophoresis, and acoustic wave-based separation. Each approach offers distinct advantages regarding separation efficiency, throughput, and device complexity. Accordingly, the literature assesses the suitability of all these methods for finger-prick blood collection and compares their implementation within microfluidic devices [1,5,14].

### 3.1. Passive Techniques for Plasma Separation in Microfluidics

Passive microfluidic techniques for plasma separation leverage the inherent properties and phenomena of fluids at microscale in precisely engineered device geometries to achieve separation without the need for external forces. These approaches rely on stable and reproducible separation mechanisms, making them highly suitable for POC applications due to their simplicity, cost-effectiveness, and reduced operational complexity. By eliminating external energy sources, such as mechanical actuation, including pumps and centrifuges, or applied electric or magnetic fields, passive methods enable seamless integration into portable and disposable (single use) diagnostic platforms. The primary mechanisms of passive plasma separation include size-based filtration, inertial separation, hydrodynamic effects, gravitational sedimentation, and paper-based approaches, each of which, or in combination, requires specific design features to maximize their effectiveness. Moreover, each category exploits distinct principles of fluid dynamics, cell deformability, or material surface/interface interactions to isolate plasma from whole blood with varying degrees of efficiency and selectivity. Design configurations and parameters, such as channel geometry, flow rate control, and material selection, are essential to ensure high plasma yield while minimizing hemolysis and cell clogging. This section provides a detailed overview of these passive strategies, highlighting low sample amounts and implementable finger-prick sample collection approaches with their working principles, typical device architectures, and relative strengths and limitations [14,15].

#### 3.1.1. Filtration-Based Techniques

The Vivid™ plasma separation membrane, a polycarbonate-based asymmetric polysulfone membrane, is widely used in microfluidic and paper-based devices due to its high plasma purity, rapid separation, low hemolysis, and compatibility with small sample volumes [16]. It enables extraction of plasma from as little as 20–50 µL of whole blood within seconds to minutes, making it suitable for finger-prick sampling and integration into POC devices. For instance, 3D-printed sample preparation devices using Vivid™ membranes demonstrated ~56.9% plasma recovery from 50 µL capillary blood in 87 s without hemolysis. Similarly, an origami-based electrochemical microfluidic paper device enabled passive plasma separation from ~20 µL finger-prick samples and multi-plexed troponin detection in ~20 min [17].

A passive microfluidic cartridge was developed that combines a gravitational-fed Vivid™ membrane separation with capillary-driven flow, eliminating the need for pumps or external actuation. A finger-prick blood volume of 10 µL is diluted in PBS-EDTA to create a working input, with the membrane capacity accommodating up to ~40 µL of diluted blood. Optical microscopy confirmed a 97% reduction in red blood cells after filtration, and spectroscopic analysis showed hemoglobin levels approaching those of centrifuged plasma, corresponding to high separation efficiency. A controlled capillary flow generating a flow rate of ~24 µL/min that sustains measurement for 12–15 min [18]. Another plasma separation microfluidic chip operates through a capillary force-driven Vivid™ membrane filtration principle. Using undiluted whole blood (hematocrit 48%), the chip consistently produced 5–30 μL of plasma within about 5 min, independent of the input blood volume. Performance validation showed a red blood cell capture rate of 99.8%, with residual RBC levels as low as 0.2%, and protein recovery rates of ~81% (total protein) and ~75% (albumin) compared to centrifugation, demonstrating strong extraction efficiency and purity. Importantly, the design achieved quantitative sampling with less than 10% error across tested ranges, making it suitable for standardized diagnostics [19].

Another microfluidic device was developed for plasma separation using capillary force-driven polysulfone membrane filtration. The main mechanism of this multi-layered PMMA-based device is the use of asymmetric bifurcating channels that leverage hydrodynamic forces to direct blood cells into collection zones while allowing cell-free plasma to flow into a collection area. The system achieves high separation efficiency, extracting 5–30 µL of plasma from 100 to 200 μL whole blood with 48% hematocrit level with 99.8% purity in 20 min [20]. Another PMMA-based microfluidic device utilized Vivid™ membrane to separate plasma from whole blood. Within these combinations of the membranes, microstructured channels in the glass fiber membrane selectively exclude blood cells, and the plasma separation membrane enables the unhindered flow of plasma to the sensing regions for analysis. Using the device, 50 μL of plasma can be separated from 200 μL of blood within 6 min and analysis for these biomarkers can be performed within 15 min [21]. Other microfluidic devices have utilized Vivid™ membranes with hydrodynamic and bifurcation effects to isolate ~96% pure plasma from 100 µL whole blood in 3 min for HIV RNA extraction, achieving 30–40% recovery [22]. Gravity-driven capillary devices combined with membrane filtration achieved ~99.5% cell-free plasma from 2.3 mL blood in 8 min, suitable for HIV detection in resource-limited settings [23].

**Figure 3:**
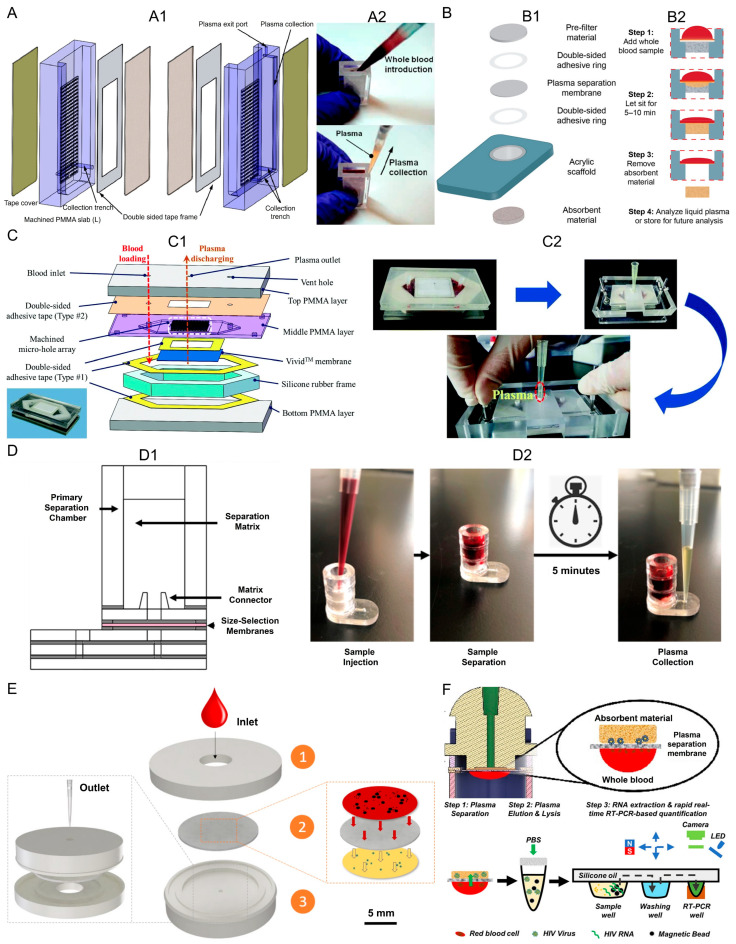
Examples of various microfluidic devices built for plasma separation through filtration mechanisms. (**A**) Using the Vivid™ plasma separation membrane, the assembly and operation of a microfluidic device with PMMA slaps and tape frames (**A1**): schematic design; (**A2**): blood loading and plasma collection) [24]. (**B**) A layered plasma separation device using a pre-filter, adhesive rings, and absorbent (**B1**): the schematic design of the device; (**B2**): workflow showing step by step separation from whole blood”, to plasma separation) [25]. (**C**) Microfluidic device using layered PMMA and a silicone rubber fold with micro-hole array for plasma separation, as shown (**C1**): schematic and design; (**C2**): experimental plasma separation example) [23]. (**D**) Size-exclusion-based plasma separation device (**D1**): chamber design and matrix connectors; (**D2**): use of device showing sample injection and subsequent 5 min retrieval of plasma) [26]. (**E**) Compact, circular separating device shown in cross-sectional design showing inlet, membrane layer, and collected plasma separated zones [18]. (**F**) Membrane−absorbent composite device to separate full blood plasma with PBS flushing [22].

The Vivid™ membrane devices yielded 275 ± 33.5 μL of plasma from 1.8 mL whole blood, all without compromising any viral particle integrity for subsequent testing, in a high-throughput PMMA-based device and in under 5 min. Other integrated devices provide on-chip separation of plasma and analysis of, glucose, cholesterol, and other components from whole blood (600 μL) [24]. Multi-layer-configured devices using pre-filter layers combined with Vivid™ membranes were able to (i) provide 65.6 ± 3.9 μL of plasma from 250 μL of whole blood; (ii) achieve 53.8% separation efficiency; and (iii) allow for the detection of IgG, IFN-γ, and HIV-1 RNA [25]. Devices with Vivid™ membranes and for immunocapture provided ~97% plasma recovery from 400 μL of whole blood with 65% hematocrit in under 5 min and allowed for detection of small molecules, proteins, and nucleic acid from plasma for use in diagnostic testing [26]. Figure 3 shows examples of different microfluidic devices for plasma separation based on filtration mechanisms.

Overall, filtration-based plasma separation devices (Table 1) demonstrate a consistent and robust strategy for extracting plasma from whole blood across a wide range of formats and sample volumes. Independent of the specific microfluidic design, the combination of asymmetric membrane filtration with passive transport mechanisms enables rapid plasma generation without external actuation. These studies collectively show that membranes support reliable integration with downstream biochemical and molecular assays while preserving sample integrity. Filtration based platforms can be viewed as a standardized and adaptable foundation for point-of-care plasma separation systems.

#### 3.1.2. Sedimentation-Based Techniques

**Figure 4:**
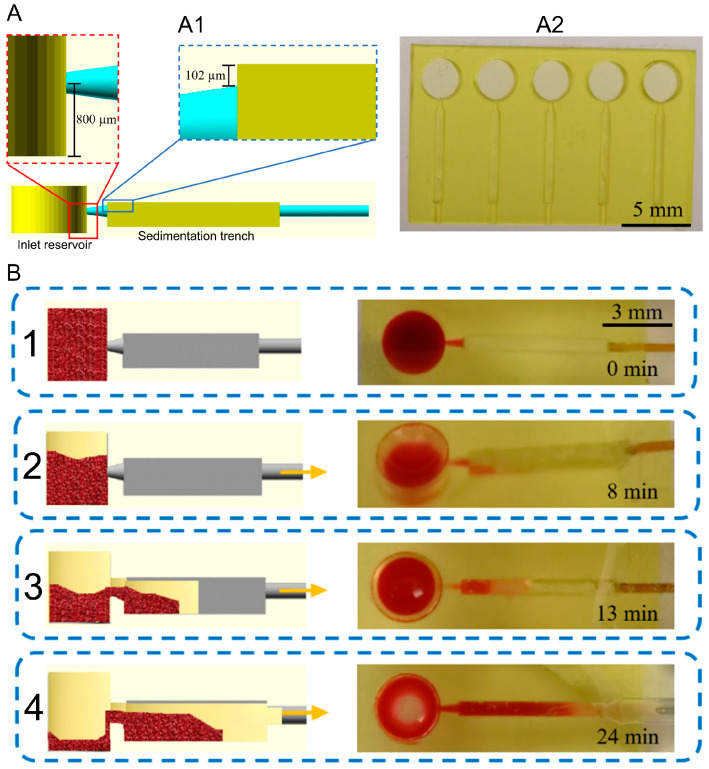
Microfluidic instrument exhibits plasma separation through sedimentation. (**A**) Device design: (**A1**) is a schematic of the connecting channel between the inlet reservoir and the sedimentation trench. (**A2**) is a picture of the fabricated chip. (**B**) Plasma separation process: (**B1**–**B4**) sequential images showing blood flow and a progressive degree of separation of plasma over [27].

This method is simple, pump-free, and can be integrated into microfluidic channels or vertical column designs for passive plasma extraction. However, separation speed and efficiency depend on hematocrit level, channel geometry, and the duration allowed for sedimentation. Since it does not provide high purity or separation efficiency, this method is generally used in combination with a membrane or another technique as a dual-assisted approach, although it is a useful technique for preliminary separation. Here, a passive, 3D-printed microfluidic platform for sedimentation-based plasma separation is introduced. Extraction relies on gravity-driven RBC sedimentation, and the device was fabricated via SLA 3D printing, using customized resins (hydrophilic and hydrophobic PEGDA-based formulations), directly yielding a monolithic microfluidic chip without multi-step assembly. As for extraction time, the protocol includes waiting 8 min for sedimentation, followed by flow at 1 µL/min for 35 min, during which plasma begins separating after about 5 min and the trench fills in roughly 16 min. The yield of plasma is modest- about 5 µL recovered from the 12 µL blood sample (Figure 4 and Table 2) [27].

While gravity-driven sedimentation is robust and easy to implement, its performance is inherently limited by hematocrit dependence and relatively long processing times. Consequently, this approach is better suited for preliminary plasma enrichment rather than standalone high-purity separation. Its true value emerges when used as a complementary element alongside membranes or other separation mechanisms.

#### 3.1.3. Passive Flow-Driven Techniques

**Figure 5:**
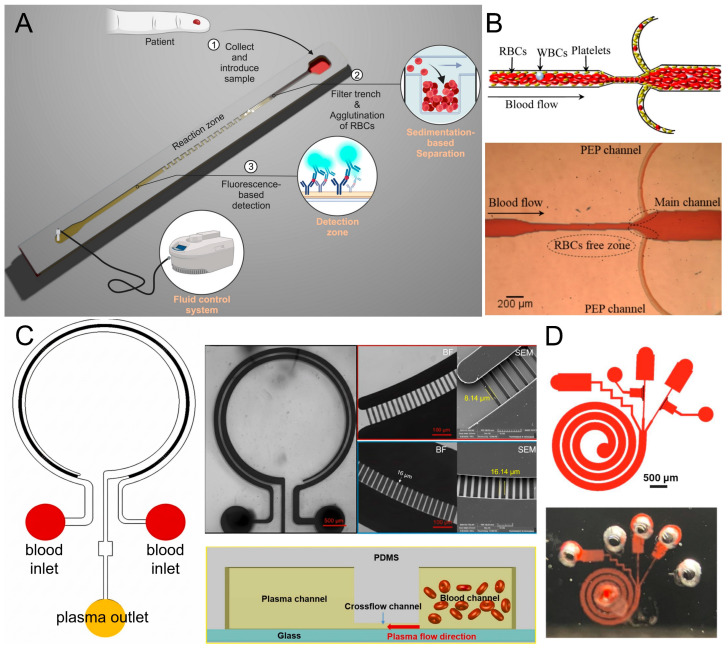
Examples of fluid-driven microfluidic devices for plasma separation. (**A**) Lateral flow assay: the sample is introduced into the inlet (1) towards the filter trench in which RBCs are separated from blood by sedimentation (2), and the plasma continues to flow to reaction and detection zones, which form the sandwich complex and capture it (3) [28] (**B**). Microdevice schematic for plasma extraction: whole blood is introduced into an inlet, and RBCs, WBCs, and platelets are separated within the microchannel. The device directs the plasma extracted product (PEP) sample to the outlet for subsequent use [31]. (**C**) Schematic illustrating microfluidic device (left) with blood, plasma, and cross-flow channels and a brightfield and SEM images of the cross-flow channels (right top) and cross-sectional view (right bottom) of plasma separated from blood using cross-flow channels [32]. (**D**) Design and experimental image of a spiral microfluidic device: the schematic design (top) represents the layout of the spiral, multi-outlet design, and fabricated device (bottom) after the plasma separation experiment [30].

Flow-driven microfiltration separates plasma passively by primarily redirecting blood through microchannels which utilize fluid dynamics to divert cells while permitting the plasma to continue along the stream. One example of this is a lateral flow chip which relies on the agglutination of red blood cells and gravity assisted with sedimentation to obtain approximately 96% efficiency which yielded 7.23 µL of plasma from 25 µL of whole blood in under 10 min [28] (Figure 5A). Another flow-driven microfluidic device’s separation principle exploits biophysical and geometrical effects such as the Fahraeus effect, Zweifach–Fung bifurcation law, inertial lift, drag forces, and constriction–expansion channel geometries to form a stable cell-free layer for plasma extraction. It processes undiluted whole blood samples across a wide hematocrit range (8.16–73.4%), with optimized flow at 0.6 µL/s. Plasma separation efficiency reached 99.97% at 54.8% hematocrit and 93.5% at 73.4% hematocrit, though plasma yield was relatively low (1–6% depending on outlet) [29].

The other systems, which are Dean vortex-based, utilize secondary flows occurring in spiral channels to concentrate the cells and separate the plasma from the blood components. A PDMS microfluidic device was utilized that combined Dean vortices with cross-flow filters to separate plasma without the use of pumps and completed the test in less than 5 min with nearly 88% removal of cells; however, the yield varied from 48% (20% Hct) to 30% (50% Hct) [30] (Figure 5D). A similar spiral-channel device could enrich platelets (8.7-fold) while still producing plasma with very low RBC concentration [31] (Figure 5B). A cross-flow device with a gradient-width yet again improved separation efficiency (>99.6%), recovery, and resin build-up mitigation at a flow rate of 2000 µL/h obtained from linear designs [32] (Figure 5C).

Methods based on geometry leverage bifurcation or serpentine designs to enhance separation. Blood modeling of bifurcation-based chips yielded numerical predictions of 64% plasma yield with 100% purity for blood with 10.4% Hct at the flow rate of 13.3 µL/h [33]. Simulation studies, in which various geometries (trifurcations, serpentine, triangular, square) were compared, suggested designs that, when optimized for yield, were theoretically able to reach up to 98% plasma yield [34]. A numerical simulation study of blood plasma separation in passive microdevices was presented, focusing on the Zweifach–Fung bifurcation law as the underlying plasma separation principle [35]. Three device geometries were analyzed as constriction-expansion channels, Tau-shaped type 1 (τ-135°), and Tau-shaped type 2 (τ-180°). These devices operate without external fields, relying solely on hydrodynamic effects, cell migration, and channel geometry to enhance the cell-free layer and direct plasma into side outlets with flow rates of 0.2–0.8 mL/min and inlet hematocrit values ranging from 0 to 45%. The separation efficiency strongly depended on hematocrit and channel design: τ-180° devices achieved 100% plasma separation up to 24% hematocrit, τ-135° reached 99.9% at 3% hematocrit, and CE designs showed 84% at 7% hematocrit. Plasma purity was also highest in the τ-180° geometry (100% up to 24% hematocrit). A straight microfluidic channel device that separates plasma and platelets from undiluted whole blood using the combined principles of cell margination and inertial focusing. The device contains nine bifurcated channels, where fluidic resistance at the outlets can be tuned to selectively divert red blood cells, platelets, and plasma. Using 1–4 mL of anticoagulated whole blood at hematocrit levels ranging from 35 to 63%, the device achieved 99.9% red blood cell removal and 97.2% platelet removal for plasma extraction, yielding about 80 μL of plasma within 10 min when operated in continuous recirculation [36]. A hybrid microfluidic electrochemical assay was proposed for cervical cancer detection combining passive plasma separation where the principle is based on a passive plasma separator that uses a 0.6 µm pore-size filter membrane coupled with parallel capillaries and a capillary micropump to isolate plasma from whole blood via capillary action and sedimentation, without external forces. From 160 to 200 µL of undiluted whole blood at physiological hematocrit (~45%), the PPS device collects about 22 µL of plasma within 10 min, achieving ~99% purity and 25% yield, with minimal hemolysis [37].

Flow-driven microfluidic plasma separation methods (Table 2) demonstrate high plasma purity, but often at the expense of limited yield and strong dependence on hematocrit and flow conditions. Dean vortex and bifurcation-based designs highlight how geometric optimization can enhance cell focusing and plasma skimming, yet no single geometry performs optimally across all operating ranges. Collectively, passive flow-driven microfiltration functions most effectively as geometry-dependent strategy that can be combined with other separation elements for diagnostic platforms.

**Table 2:** Overview of sedimentation-based and passive flow-driven plasma separation methods and performance parameters.

Reference	Sample Volume	Extraction Efficiency	Yield	Blood Sample	Extraction Time	Hematocrit Level	Final Purpose
I-Sedimentation-based method							
[27]	12 μL	NA	~5 µL	Undiluted and diluted blood	35 min flow + 8 min pre-sedimentation	NA	Comparison between different 3D designs for plasma separation
II-Passive Flow-Driven methods							
[28]	25 μL	96%	7.23 μL	Whole Blood	10 min	44%	NT-proBNP detection
[29]	NA	(99.97–93.5%)	1–6%	Whole Blood	NA	(8.16–73.4%)	Colorimetric detection of creatinine and urea
[30]	2–5 μL	88%	NA	Whole Blood	<1 min	30–45%	Extraction of the plasma with lower hematocrit
[32]	Cont. System	97% for RBCs	28%	Whole Blood	2000 μL/h	NA	plasma separation
[33]	Simulation	~64%	25–35%	Whole blood	NA	10.4%	Optimization of chip geometry capable of efficiently separating plasma
[34]	Simulation	NA	98% (in theory)	Whole Blood	NA	NA	Plasma-separating microchannel design and simulation
[35]	NA	84–99.9%	NA	Whole Blood	0.8 mL/min	0–45%	Observation of Fahraeus effect, the Fahraeus–Lindquist effect, and the Zweifach–Fung effect on plasma separation
[36]	NA	99.9% for RBC, 95.4% for PLTs	80 μL	Whole Blood	10 min	NA	Platelet or plasma separation
[37]	160 μL	99%	22 μL	Whole Blood	10 min	45%	Cervical cancer detection

NA—Not applicable.

#### 3.1.4. Superhydrophobic Membrane

**Figure 6:**
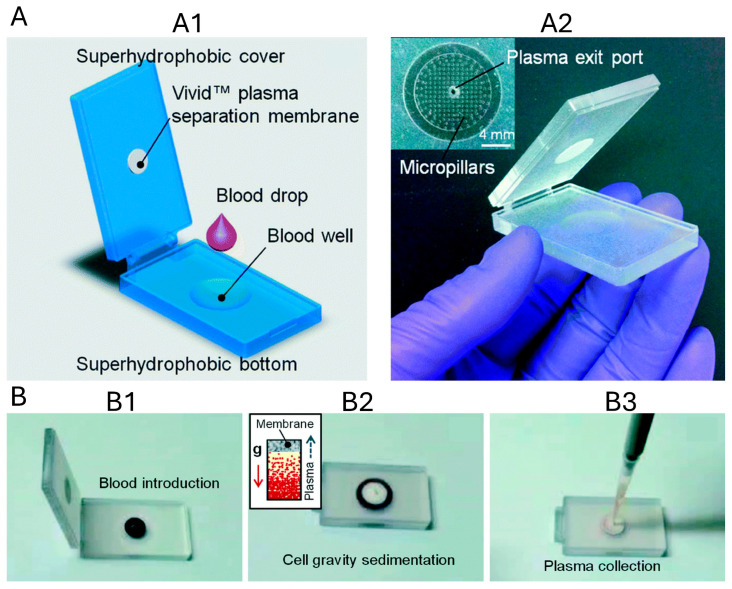
Superhydrophobic device for blood plasma separation from whole blood. (**A**) Device layout including a superhydrophobic cover, superhydrophobic bottom, blood well, and plasma separation membrane (**A1**), and a photo of fabricating prototype with plasma exit port and micropillar (**A2**). (**B**) Device operation includes inserting blood (**B1**), gravity driven cell sedimentation of blood plasma through membrane (**B2**), and collecting plasma with pipette (**B3**) [38].

A clamshell-style, superhydrophobic membrane-based plasma separator was developed for finger-prick blood collection and plasma separation (Figure 6) [38]. It comprises a superhydrophobic top cover and bottom substrate sandwiching a Vivid™ membrane, designed to keep the blood film thin and contracted for optimal processing. The superhydrophobic surfaces promote high contact angles, reduce biomolecular adhesion and improve sample integrity. The device passively extracts ~65 ± 21 μL of plasma from 200 μL of whole blood in under 10 min. When blood was spiked with Schistosoma mansoni genomic DNA, the system achieved >84.5 ± 25.8% recovery, indicating successful capture of genetic biomarkers.

Another superhydrophobic membrane was used for plasma separation that combines Janus filtration with RBC agglutination. The plasma separation principle relies on antibody-induced RBC aggregation to form large cell clusters that are blocked by the micropores of a Janus titanium membrane, while plasma spontaneously flows from the hydrophobic to the hydrophilic side via capillary and wetting gradient forces. Using 10–100 µL of whole blood, the system achieves plasma yields of ~80% with 99.99% purity in 20–80 s. The platform is robust across a wide hematocrit range (15–85%), with no significant drop in yield or purity, and it prevents hemolysis. Depending on the input, plasma recovery volumes reached up to ~85% of the available plasma fraction [39].

Superhydrophobic membrane-based plasma separators (Table 3) demonstrate how surface wettability engineering enhances passive plasma extraction. By combining superhydrophobic interfaces with membrane filtration or Janus wetting gradients, these systems maintain thin blood films, reduce fouling, and preserve sample integrity. The addition of sedimentation or RBC agglutination further stabilizes separation performance without external actuation. Overall, superhydrophobic membrane platforms represent a robust and scalable strategy for plasma separation in high-volume diagnostic applications.

#### 3.1.5. Paper-Based Techniques

**Figure 7:**
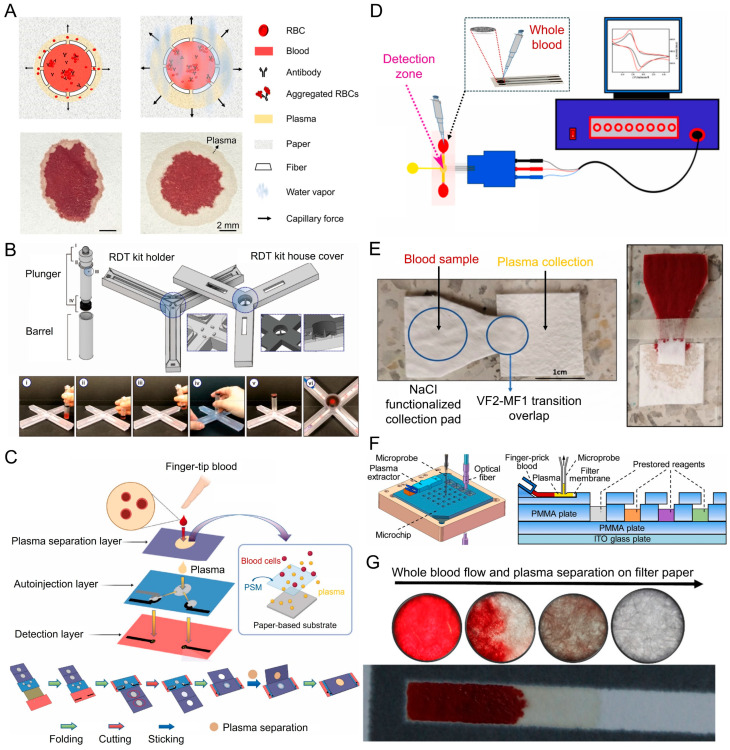
Paper-based techniques for plasma separation. (**A**) Shows the mechanism for plasma separation utilizing antibody-mediated RBC aggregation to capitalize on capillarity and to enable the migration of plasma from the defined blood application zone to a clear plasma ring [40]. (**B**) Displays an RDT holder with a plunger and protective housing for initiating delimited plasma separation and subsequent plasma transfer [43]. (**C**) Provides a multi-layered paper-based device that separates plasma, auto-injection, and detection layers designed for finger-stick device diagnostic blood processing and downstream analysis [45]. (**D**) Shows a hybrid paper–microfluidics platform that separates plasma and connects to an electronic detection system [41]. (**E**) Illustrates a pragmatic paper-based approach with two independent blood application and plasma separation zones suitable for implementation into ELISA immunoassays [42]. (**F**) Presents a microchip-assisted method of plasma separation utilizing a filter membrane and a PMMA plate for optical-based analysis [44]. (**G**) Photograph depicts paper-based device that demonstrates visible plasma separation from blood directly observable in a housing and plasma migration within a hydrophobically to the zone along the paper strip [46].

A humidity-enhanced paper-based microfluidic device for plasma separation using Chinese Xuan paper pre-treated with blood typing antibodies. The plasma separation principle combines antibody-induced RBC agglutination with capillary-driven wicking, where high environmental humidity further improves the separation by increasing the difference in wicking distance between plasma and RBC aggregates (Figure 7A). Only 10–40 µL of whole blood is needed, and plasma can be separated within 5 min. The method achieved up to 60.1% plasma yield from a 10 µL blood sample with 99.99% purity. The system was validated across hematocrit levels from 30% to 60%, demonstrating versatility for a wide range of clinical samples. Protein and glucose recovery rates were approximately 80%, confirming suitability for downstream assays [40].

A biodegradable electrochemical microfluidic paper-based device that integrates plasma separation and non-enzymatic detection of ascorbic acid in a single step (Figure 7D). The plasma separation principle relies on a layered structure of blood separation membrane combined with filter paper, fabricated via wax-dipping technique to wick plasma away from RBCs through capillary forces. Using 80 µL of undiluted whole blood at physiological hematocrit levels (27–55%), the device successfully delivered plasma to the detection zone within 200 s [41].

A paper-based microfluidic blood plasma separation device was designed for biomarker detection, specifically targeting S100B protein for traumatic brain injury diagnostics (Figure 7E). The plasma separation principle combines osmotic pressure, size-exclusion by filter membranes, and capillary-driven lateral–vertical flow, where a functionalized pad induces RBC crenation and aggregation, followed by plasma wicking into a detection pad. Using 300 µL of whole blood at 45% hematocrit, the device consistently yielded 50 µL of plasma in about 3.5 min with a separation efficiency above 95% [42].

A self-pressure-driven lateral flow plasma separation device was developed, based on the principle of manually pressurizing whole blood through a glass fiber filter membrane that captures RBCs and other hematocytes while allowing plasma to pass through. The device features include a compact 3D-printed barrel-and-plunger design, with integrated seals and filters, connected directly to multiple lateral flow assay strips for immediate biomarker detection (Figure 7B). Only a few microliters of whole blood are required, and plasma can be extracted in 30 s to 1 min [43].

An integrated microfluidic system for multi-target biochemical analysis was presented that incorporates filter membrane-based plasma separation with automated liquid handling. The plasma separation principle relies on lateral flow through a filter membrane, where RBCs are trapped in the micropores and plasma is aspirated by a microprobe under controlled negative pressure (Figure 7F). Using a single drop of whole blood (~30 µL), the system achieves quantitative plasma extraction of ~5–7 µL within 30–75 s. The method works robustly for hematocrit levels between 30 and 50%, with extracted plasma volumes showing a negative correlation to hematocrit [44].

A self-driven origami paper-based electrochemical microfluidic chip that integrates plasma separation and biomarker detection. The plasma separation principle relies on an asymmetric polysulfone plasma separation membrane combined with capillary-driven flow in the paper substrate, which traps blood cells while allowing plasma to pass into the detection region. The system requires ~73.3 µL of whole blood and achieves plasma separation in 75 s, with a blood cell removal efficiency of 99.91% and a plasma recovery yield above 80% [45].

A paper-based plasma separation assay presented that uses a universal anti-H agglutinating antibody to enable extraction of plasma from whole blood. The plasma separation principle combines antibody-induced RBC agglutination with passive filtration through filter paper, where aggregated RBCs are trapped in the paper fibers while plasma wicks forward (Figure 7G). Using only a 7 µL drop of whole blood, the device achieved 72% plasma separation efficiency in a few minutes, with 99.99% purity. Plasma separation was validated across hematocrit levels averaging 39–40%. The approach successfully separated plasma from 116 out of 119 donor samples (97.5% success rate), and biomarker recovery tests showed accurate detection with ~107% recovery [46].

Paper-based plasma separation platforms (Table 3) offer clear advantages in simplicity, low cost, and rapid operation, making them well suited for decentralized testing. By leveraging capillary wicking, membrane filtration, and antibody-induced RBC agglutination, these systems can achieve high plasma purity using small blood volumes and without external instrumentation. However, their performance is often sensitive to hematocrit, environmental conditions, and paper or membrane variability, which can limit reproducibility and quantitative control. Yield is also constrained in some designs, particularly when rapid separation or minimal sample input is prioritized. Overall, paper-based plasma separation represents a practical and scalable solution for rapid screening and integrated assays, but it is best applied where ease of use and accessibility outweigh the need for precise volumetric accuracy.

### 3.2. Active Techniques for Plasma Separation in Microfluidics

Active microfluidic plasma separation techniques employ externally applied forces to manipulate cellular and fluidic components within blood, enabling efficient isolation of plasma on a miniaturized platform and integrating external fields to achieve higher selectivity, tunable operation, and improved control over separation outcomes [47]. These techniques exploit a variety of physical phenomena, including flow-driven and pump-assisted, centrifugation, electrical, and acoustic, to selectively displace or trap blood cells while allowing the cell-free plasma to be collected. Together, these active strategies represent powerful alternatives to conventional blood processing methods. Each modality offers unique strengths in terms of throughput, plasma yield, and integration potential, while also presenting challenges such as energy requirements, device complexity, and scalability.

#### 3.2.1. Active Pump-Assisted Techniques

Active pump-assisted plasma separation microfluidic devices rely on externally applied pressure or syringe pumps to control fluid dynamics and direct blood through specialized channel geometries or filtration units [48]. By precisely tuning flow rates and shear stresses, these systems can guide plasma and cellular components along distinct paths, often exploiting inertial effects or hydrodynamic focusing for separation. Although they require external equipment, their adaptability to different flow regimes allows high throughput and scalability.

**Figure 8:**
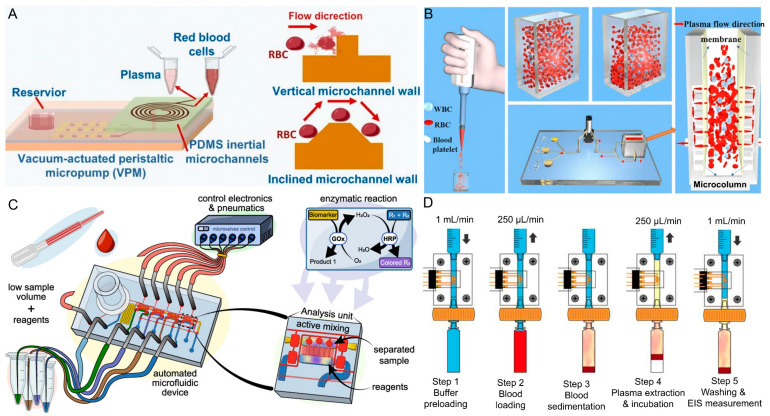
Flow-driven and pump-assisted microfluidic techniques for plasma extraction from whole blood. (**A**) Vacuum-actuated peristaltic micropump (VPM) integrated with PDMS inertial microchannels enabling plasma cell separation through inclined and vertical channel walls by vacuum-driven flow [49] (**B**) Membrane-based microcolumn system using syringe-driven flow to separate plasma from whole blood via controlled filtration, enabling efficient plasma collection for downstream applications [50]. (**C**) Automated pneumatic microfluidic device with negative pressure-based plasma separation and integrated bioanalysis modules for low-volume blood samples, supporting fully automated workflows [56]. (**D**) Syringe pump-assisted hybrid microfluidic system combining sedimentation and electrochemical sensing for on-chip plasma extraction and in situ biomarker detection: system layout and stepwise operational sequence showing buffer loading, blood sedimentation, plasma extraction, and measurement [57].

One of the pump-assisted active plasma separation devices is a vacuum-actuated peristaltic micropump that was integrated with inertial microfluidics to achieve rapid plasma separation. The separation principle relies on combining peristaltic pumping with Dean vortices in spiral microchannels to direct RBCs away from the plasma stream (Figure 8A). Only a 5 µL of whole blood sample had been used and diluted with different ratios to determine the extraction efficiency. Plasma extraction efficiency reached 98.5 percent, with yields strongly influenced by dilution ratio: efficiencies of 92.7, 96.5, and 98.8 percent were obtained at 20×, 40×, and 90× dilutions, respectively. The system produces hemolysis levels around 2% when using inclined channel walls, indicating that the extracted plasma maintains high quality. The separation time is under 1 min with diluted sample [49].

A vertical placement of an asymmetric polysulfone membrane forms the core of the plasma separation system, where gravity assists cell sedimentation while plasma is drawn perpendicularly through the membrane (Figure 8B). The setup is built with a micro-pillar array supporting the membrane, a micropump to drive fluid flow, and a compact detection chamber. In operation, 0.6 mL of whole blood is introduced, followed by a short 5 min standing period to allow RBCs to settle before plasma is pushed across the membrane. The design maintains high separation efficiency with negligible hemoglobin release and delivers plasma suitable for biochemical assays with yields around 100 µL per run [50].

Another microfluidic device was developed that employs gravity sedimentation in a multi-trench design to separate platelet-rich plasma from whole blood, with a syringe pump used to deliver the sample onto the trenches. The plasma separation principle is based on the density difference between blood cells and plasma, with red and white blood cells sedimenting into the trenches while platelets remain in the plasma fraction. Three trench geometries (rectangular, circular, and large trenches) were tested, each integrated into a microchannel system with an on-chip reservoir for 1 mL of whole blood input. The device was validated with undiluted whole blood at physiological hematocrit levels (45–55%), demonstrating robust sedimentation dynamics. Using optimized flow conditions (12.5–25 µL/min with a syringe pump), the system produced up to 250–300 µL of platelet-rich plasma in 40–65 min, corresponding to a 25% yield of the input sample, significantly higher than the 10–15% typically achieved with centrifugation. The separation efficiency achieved ~99% RBC removal, up to 94% WBC removal (geometry-dependent), and ~93% platelet-rich plasma purity with a 2.5-fold increase in platelet concentration, while limiting platelet activation to ~13% compared to ~31% in centrifugation [51].

A microfluidic platform for extracting platelet-free plasma using a peristaltic pump for both creating flow by pressure and vacuum effects was designed to support downstream analysis of microRNAs and extracellular vesicles. The system processes diluted blood samples with PBS (1:1 ratio) with volumes of 3-milliliters. The device enables higher throughput of 0.1 mL of blood per minute in its system, which allows the processing of a standard 3 mL blood tube within 30 min. The device can directly handle samples with a hematocrit of around 25%. In terms of performance, the device demonstrated platelet removal efficiency of over 99.9%, generating high-purity plasma suitable for sensitive biomarker detection [52].

A microfluidic device focuses on enhancing blood plasma separation in different shaped, PDMS-based channels through surface modification to improve wettability and reduce issues such as air bubble trapping and cell aggregation. The plasma separation principle is cross-flow filtration created by a syringe pump and supported by multi-stage pillar arrays and hydrophilic surface treatment. Blood samples were prepared from healthy volunteers, diluted to 2% hematocrit in physiological salt solution, and processed at flow rates of 50–200 µL/min using syringe pumps. Each test used a 5 mL blood sample volume, with outlet fractions collected in microtubes in 20 min [53].

An integrated microfluidic system was designed that combines a pneumatic peristaltic micropump with spiral inertial microchannels for plasma separation. The separation principle is based on inertial spiral microchannels, where shear-gradient lift and wall lift forces in trapezoidal spiral channels focus and direct blood cells away from plasma, allowing clean plasma collection. A key feature of the device is its triple pneumatic peristaltic micropump design, capable of generating extremely high flow rates up to 3510 µL/min and high backpressure without leakage, ensuring efficient on-chip pumping without external syringe pumps. The device requires only 4 µL of whole blood, collected via a fingertip lancet, which is then diluted 45 times with PBS (total 180 μL) before processing. Plasma extraction efficiency reached approximately 97% after four rounds of recirculation, with the entire process completed in less than 3 min [54].

A flow-rate-insensitive plasma extraction device was reported based on a spiral microchannel with ordered micro-obstacles that stabilize and accelerate Dean-like secondary flows by syringe pumping. Using diluted blood to 1% hematocrit, the system achieved 99.70% rejection of all blood cells including platelets, with a plasma recovery yield of 67.57% at optimal conditions. The device can process samples in the range of 1 to 2.5 mL/min flow rates, maintaining stable focusing and separation efficiency for up to 60 min of continuous operation [55].

A portable plasma separation device with a pneumatic control system was developed that drives a valve-enabled PDMS microfluidic chip which separates plasma by passing whole blood through a plasma separation membrane placed at the sample inlet while applying negative pressure to draw cell-free plasma into a serpentine collection microchannel, while blood cells are retained on the membrane (Figure 8C). The device has mini diaphragm pumps, digital pressure sensors, a microcontroller and multiple solenoid valves and manifolds that generate both high pressure for microvalve actuation and low pressure for fluid handling, plus firmware routines for valve sequencing, degassing and active mixing into four analysis units with 50 nL reagent and sample chambers. The sample volume is 8 µL of whole blood deposited onto the membrane for the automated workflow; in the assay protocol plasma is pulled through the membrane with negative pressure for about 30 s and collected plasma capacity is roughly 1 µL. The device integrates plasma preparation and glucose detection by using blood plasma into a single disposable platform [56].

A microfluidic platform with an integrated miniaturized electrochemical sensor for combined on-chip plasma extraction and in situ detection of C-reactive protein. The plasma separation principle is a hybrid approach that uses sedimentation to accelerate RBC settling, followed by membrane filtration with a syringe assisted flow system (Figure 8D). From 400 µL of undiluted whole blood, the platform consistently yielded 100 µL of plasma within 7 min. The system worked robustly across hematocrit levels up to 50%. The entire workflow, including plasma extraction and biomarker incubation, was completed in about 40 min [57].

Active pump-assisted plasma separation techniques (Table 4) offer precise control over flow conditions, enabling high separation efficiency, throughput, and compatibility with complex downstream analyses. By exploiting inertial effects, Dean flows, cross-flow filtration, and controlled sedimentation, these systems can deliver highly purified plasma with reproducible performance. However, their dependence on external pumps, pneumatic control, and auxiliary hardware increases system complexity, cost, and power consumption. Many implementations also require blood dilution or multi-step operation, which can limit usability in decentralized settings. As a result, pump-assisted platforms are best suited for laboratory, clinical, or automated diagnostic environments where performance and scalability outweigh simplicity.

#### 3.2.2. Centrifugation-Based Techniques

Centrifugation-based active plasma separation microfluidic devices employ rotational forces to drive blood components radially outward according to their density, enabling rapid and efficient plasma extraction from whole blood [58]. These systems typically integrate microchambers or spiral channels within a compact lab-on-a-disk format, where spinning generates controlled separation without the need for external pumps. They can handle relatively large sample volumes compared to other microfluidic methods. Centrifugation microfluidics straightforward operation and compatibility with point-of-care platforms highlight their potential for routine medical testing.

**Figure 9:**
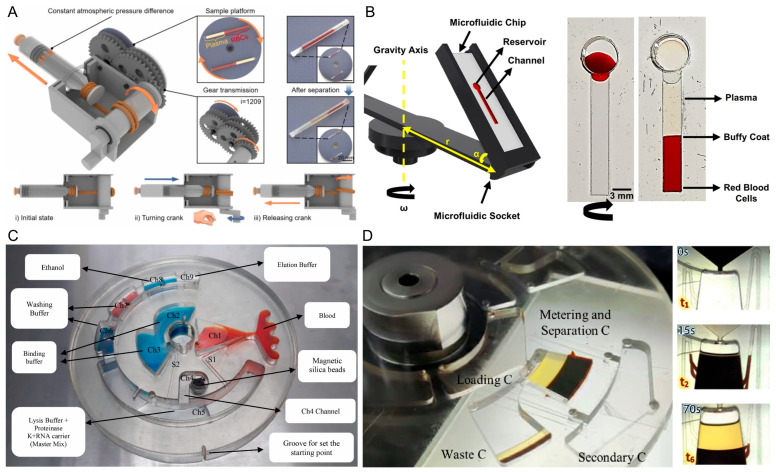
Plasma separation utilizing centrifugation-based methods. (**A**) Atmospheric pressure-driven centrifuge designed with a sealed syringe coupled with a gear-driven transmission mechanism to produce a stable (and reproducible) rotational force for plasma separation; the apparatus spins at a stable ~2300 rpm and achieves approximately ~93% plasma purity [59]. (**B**) A microfluidic platform (the Spinochip) that utilizes centrifugal forces to separate whole blood into layers of plasma, buffy coat, and red cells in a dead-end channel of a microfluidic system; schematic illustrating centrifugal-driven plasma displacement from whole blood into the microfluidic channel, resulting in collection of the plasma layer by the end of a 10 min centrifugation at 4000 rpm [60]. (**C**) A fully automated centrifugal lab-on-a-disk method using fully automated, process-consuming plasma separation and extraction of nucleic acid using magnetic silica bead technology; key reagents (lysis, binding, washing, elution buffers) are delivered in separate channels for common cffDNA extraction application from 3 mL maternal blood [61]. (**D**) A unique centrifugal microfluidic disk that has been developed with specific siphon valves, which provide hematocrit-independent separation of plasma; the disk consists of a multi-layered configuration and optimized loading, metering, and separation, and waste chambers that utilize a unique dip (siphon) geometry to consistently achieve >99% plasma purity and yield [63].

For instance, uniform plasma extraction was achieved through an atmospheric pressure-driven centrifuge that converts vacuum release inside a syringe into constant rotational force, ensuring reproducible separation. The design incorporates a sealed syringe, a gear transmission system, and a sample platform capable of holding microscale blood tubes, providing a stable spin at ~2300 rpm regardless of the operator (Figure 9A). Only 15 µL of whole blood is required and the setup reached ~93% plasma yield and ~99.99% purity within 250 s, supported by the Boycott effect to accelerate cell sedimentation. The approach works across a hematocrit range of 10–60%, and a calibrated ruler integrated into the platform allows direct hematocrit measurement [59].

Another centrifugal microfluidic system, the Spinochip platform, was introduced to exploit dead-end channel filling via spinning to achieve on-chip plasma separation together with hematocrit and white blood cell analysis. The principle relies on centrifugal forces that stratify whole blood into plasma, buffy coat, and red cell layers, while compressed air is expelled from the channel during rotation to allow controlled liquid entry and separation without valves (Figure 9B). Only 10 µL of whole blood is required per test, and under optimized conditions (4000 rpm, 10 min), the chip provides hemolysis-free plasma that can be collected directly from the channel with analysis completed in about 10 min. The device reliably separates samples at physiological hematocrit values (~45%), and was validated against clinical hematocrit and WBC measurements, showing strong correlation [60].

A fully automated centrifugal microfluidic lab-on-a-disk system was designed for isolating cell-free fetal DNA (cffDNA) from whole blood. The plasma separation principle is based on centrifugal forces that sediment RBCs while transferring plasma through siphon valves into designated chambers, followed by DNA binding to magnetic silica beads which are displaced between reagent chambers using external magnets (Figure 9C). The system processes 3 mL of maternal whole blood at 45% hematocrit, yielding 1.3 mL of highly pure plasma (99.95%) within the device. The plasma is then used for DNA extraction, where the total cffDNA separation and purification is completed in about 20 min. The plasma yield is controlled by siphon design, ensuring consistent recovery of 1.3 mL suitable for downstream analysis [61].

Another centrifugal microfluidic lab-on-a-disk system was designed and evaluated for efficient blood plasma separation and transfer, combining numerical modeling and experimental validation. The system processes 100 µL of whole blood per separation chamber. Numerical and experimental results confirmed over 90% plasma separation efficiency and up to 95% transfer efficiency of separated plasma without significant cell contamination. The yield of recovered plasma was geometry-dependent, with optimized siphon designs transferring ~26–36 µL of plasma from an initial 70 µL sample after separation. Tests were conducted with whole blood at physiological hematocrit (45%), and separation was achieved within 8 min, depending on geometry and rotation protocol [62].

A hybrid siphon valve design integrated into a centrifugal lab-on-a-disk platform was introduced for hematocrit-independent plasma separation from whole blood. The device features include a multi-layer PMMA disk with pressure-sensitive adhesives and glass coverslips used as super-hydrophilic surfaces, which eliminate the need for surface treatments and ensure siphon actuation (Figure 9D). Each disk contained a loading chamber, main reservoir, waste chamber, secondary chamber, and four siphon valves, each calibrated to operate at different hematocrit levels (20–50%). The system processed 2 mL of whole blood per test for about 5 min to complete separation. It was demonstrated plasma purity greater than 99% with a 75% increase in plasma yield compared to conventional single-valve approaches, particularly benefiting samples with low hematocrit (<20%) [63].

A centrifugal microfluidic system integrated with Alphalisa immunoassay was introduced for the one-step detection of pepsinogen and detection of gastric cancer biomarkers directly from whole blood. The plasma separation principle is based on an innovative “interconnected-collecting chambers collinear with rotational center” design, which stabilizes sedimentation and minimizes hematocrit effects while allowing siphon-based plasma extraction. The system requires only 50 µL of whole blood, with no need for dilution, and achieves plasma separation efficiency of ~99.9% across hematocrit levels from 12% to 48%. The plasma yield is sufficient to support downstream immunoassays, and separation is completed in about 50 s, with the full immunoassay process finalized in under 12 min. The compact chip integrates quantification chambers, siphon channels, and aliquoting chambers to ensure accurate metering, mixing, and distribution of plasma with reagents [64].

Another centrifugal lab-on-a-disk system was developed that integrates blood plasma separation and fluid reciprocation for enhanced biosensing sensitivity which relies on centrifugal sedimentation of RBCs while plasma flows into a reciprocation chamber. Only 70 µL of whole defibrinated bovine blood is required, and the system completes RBCs sedimentation and separation in 4 min with high purity and yield (99.9%) and the whole assay completed in 7 min [65].

A centrifugal microfluidic lab-on-a-disk system employed a separator gel to achieve plasma separation from whole blood. The plasma separation principle is based on centrifugal sedimentation of blood cells, with the gel positioned between the buffy coat and plasma to prevent remixing during high-speed spinning. The device features siphon-based passive valves and separation chambers capable of handling up to 3 mL of whole blood at physiological hematocrit levels (45%). The Gel-Disk achieved 99.992% plasma purity, with ~90% of the separated plasma successfully transferred to the next chamber via the siphon valve. By comparison, a NoGel-Disk design without separator gel yielded only 60% plasma transfer efficiency and lower purity [66].

Centrifugation-based microfluidic plasma separation platforms (Table 5) offer rapid, high-purity plasma extraction with strong tolerance to physiological hematocrit variations. Their ability to process larger sample volumes and integrate valving, metering, and downstream assays make them particularly attractive for automated and quantitative diagnostics. The controlled rotational actuation enables reproducible separation without complex fluidic routing. However, these systems require rotating hardware, precise mechanical alignment, and disk-specific instrumentation, which increases device complexity and limits full miniaturization. Consequently, centrifugal microfluidic approaches are well suited for laboratory and near patient testing but are less ideal for low-cost or fully disposable POC applications.

#### 3.2.3. Dielectrophoresis

Dielectrophoresis (DEP)-based active plasma separation microfluidic devices exploit the movement of blood cells in non-uniform electric fields, allowing selective manipulation of cells due to differences in dielectric properties between plasma and cellular components [67]. By applying alternating current fields through patterned microelectrodes, red blood cells and other formed elements can be trapped, deflected, or repelled while cell-free plasma flows through designated outlets. This technique enables label-free, reagent-free separation with high precision and minimal dilution. DEP-based devices are particularly effective for small-volume samples, achieving rapid plasma extraction suitable for integration with downstream biosensing or nucleic acid amplification assays.

**Figure 10:**
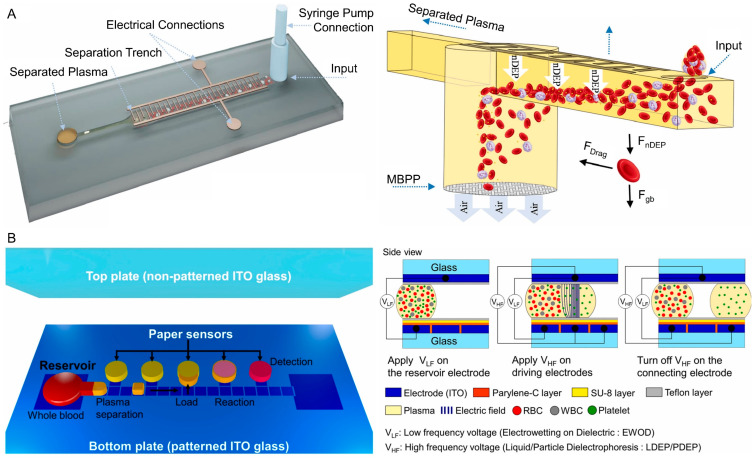
Dielectrophoretic microfluidic systems designed for plasma separation. (**A**) Diagram illustrating the main components of the device (left) and schematic diagrams of the electrode arrangement and blood cells in the microsystem (right). Cells are directed downward by negative dielectrophoretic (nDEP) forces, and into a separation trench sealed with microporous melt-blown polypropylene (MBPP) layer [68]. (**B**) The schematic of the digital microfluidic device (left) illustrates plasma droplets separated from whole blood via dielectrophoresis, while the working principle of the device is shown in the side view (right) [69].

The development of a microfluidic device for plasma separation reported that it integrates gravitational sedimentation with negative DEP to enhance separation speed and purity. The system requires only a 10 µL drop of undiluted whole blood, making it suitable for finger-prick sampling. At optimal conditions (20 V_pp_, 70 nL/s flow rate), the device achieves 99.98% ± 0.02% plasma purity, with hemolysis levels as low as 0.0015%, indicating non-hemolyzed plasma. The device was validated across a wide hematocrit range of 15–65%. The plasma yield is about 2.2 µL from each 10 µL blood sample, and the total processing time is under 4 min (Figure 10A) [68].

A digital microfluidic DEP–paper hybrid device was developed for integrated plasma separation and lithium-ion detection in whole blood. The plasma separation principle combines electrowetting-on-dielectric and DEP, which drive red blood cells away from droplets and isolate plasma droplets of 20–400 nL directly from 5 μL undiluted whole blood (Figure 10B). Only 5 µL of whole blood is required, from which five plasma droplets (about 1 µL total) can be sequentially separated and analyzed. Tested with samples at a physiological hematocrit (~45%), the device maintained stable separation without hemolysis. The platform achieves plasma separation efficiency above 90%, with negligible contamination by red or white blood cells or platelets, and produces droplets in less than 4 min [69].

DEP-based plasma separation platforms (Table 6) provide a highly selective, label-free approach for isolating plasma from very small volumes of whole blood. The ability to integrate DEP with digital or hybrid microfluidic platforms further supports precise handling and direct coupling to downstream analytical assays. However, DEP devices require patterned electrodes, electrical control systems, and careful field optimization, which increases fabrication and operational complexity. In addition, plasma yield is typically limited, making these platforms most suitable for microscale, high-precision diagnostic applications rather than bulk plasma processing.

#### 3.2.4. Acoustic Wave-Based Techniques

Acoustic wave-based active plasma separation microfluidic devices utilize surface acoustic waves or bulk acoustic waves to generate pressure nodes within microchannels, where blood cells are driven laterally by acoustic radiation forces while plasma continues to flow [70]. This active method reduces mechanical stress and minimizes hemolysis, preserving the quality of both plasma and cellular components.

**Figure 11:**
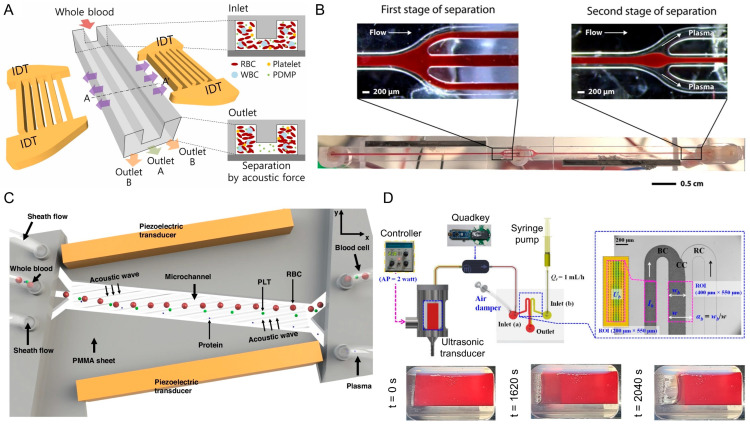
Microfluidic platforms based on acoustic waves use both bulk and surface acoustic waves for label-free plasma separation from whole blood. In (**A**), a surface acoustic wave (SAW) device with interdigital transducers (IDTs) generates acoustic radiation forces that can move red blood cells (RBCs), white blood cells (WBCs), and platelets (PLTs) laterally, allowing cell-free plasma to be extracted from collection outlets [71]. (**B**), RBCs, WBCs, and PLTs are pulled into side channels where further blood cell separation occurs before cell-free plasma is collected from the center outlet in a two-step separation [72]. (**C**) is an acoustofluidic setup using tilted piezoelectric transducers to generate standing acoustic waves within a microchannel, causing blood cells to be pulled laterally and allowing for continuous collection of cell-free plasma with minimal hemolysis [73]. (**D**) depicts a microfluidic system based on ultrasonic transducers that separates plasma while measuring viscosity. The experimental system contains a syringe pump and transducer chamber, where RBC aggregation and sedimentation takes place over 10–25 min with acoustic excitation, and cell-free plasma is directed through a co-flowing microchannel for viscosity analysis [75].

A surface acoustic wave microfluidic device was designed for the separation of plasma and platelet-derived microparticles from whole blood. The plasma separation principle is based on acoustic radiation forces generated by interdigital transducers on a lithium niobate substrate, which laterally displace RBCs and platelets toward channel sidewalls, leaving platelet-poor plasma and platelet-derived microparticles to be collected at the central outlet. The system processed whole blood with 40% hematocrit, injected at flow rates of 10–40 μL/min, with optimal performance achieved at 30 μL/min. Under these conditions, the device produced approximately 100% pure plasma with 55.6% recovery yield. Separation occurred within milliseconds (∼250 ms), enabling continuous flow operation (Figure 11A) [71].

A two-stage acoustophoresis-based microfluidic device was designed to provide cell-reduced plasma directly from whole blood with the aim of minimizing blood loss in vulnerable patients such as preterm infants. The plasma separation principle relies on ultrasound-generated standing waves that laterally migrate RBCs, WBCs, and platelets toward the channel centerline, allowing plasma to be collected at side outlets after sequential focusing and removal stages. The system processed whole blood from healthy donors, operating at a flow rate of 115 µL/min to yield plasma at 23 µL/min. The acoustophoresis method removed >99.99% of RBCs and WBCs with 90.4% platelet removal efficiency, producing plasma with cell-free hemoglobin (0.0–0.2 g/L). Plasma preparation, from collection to separation, was completed within 1 h of blood draw, significantly reducing sample handling time (Figure 11B) [72].

An acoustofluidic device that enables the automated separation of platelet-reduced plasma from whole blood was developed using a combination of acoustic forces and impedance mismatch-assisted tilted-angle pressure nodes. The system processes undiluted human whole blood at flow rates of 10–25 µL/min under low driving voltages, achieving above 90% removal efficiency of red blood cells, white blood cells, and platelets. The device has sufficient plasma yield for downstream molecular assays, with high antibody retention rates (IgG recovery), demonstrating minimal protein loss (Figure 11C) [73].

A surface acoustic wave-based microfluidic device was introduced whose principle relies on acoustic radiation forces that laterally displace RBCs and platelets toward channel walls, leaving a focused plasma stream in the center for collection. Experiments were conducted with 3.5 mL diluted blood (1:5 in PBS), with flow rates up to 50 µL/min and throughput reaching 888,000 cells/s. The device achieved RBC removal purity of 99.96% and platelet separation purity of 58.42%, with no detectable hemolysis [74].

A microfluidic platform was introduced that combines an ultrasonic transducer with a co-flowing microchannel to achieve both plasma separation and viscosity measurement. The plasma separation principle relies on ultrasonic standing waves that aggregate RBCs through acoustic and Bernoulli forces, followed by sedimentation, leaving plasma isolated within the chamber. Approximately 5 mL of blood suspended with PBS was prepared with plasma at different hematocrit levels (20%, 30%, and 40%) for testing. Plasma separation occurred within the ultrasonic chamber for over ~25 min, after which plasma was supplied to the co-flowing channel for measurement (Figure 11D) [75].

Acoustic wave-based plasma separation techniques (Table 6) enable contactless and gentle manipulation of blood components, resulting in minimal hemolysis and preservation of plasma quality. They can process undiluted blood and selectively remove multiple cellular components which makes them attractive for sensitive clinical and molecular applications. On the other hand, acoustic platforms require specialized substrates, transducers, and signal control electronics, which increase fabrication complexity and cost. In addition, throughput and device footprint can be limiting factors for point-of-care deployment. Acoustofluidic plasma separation is well suited for high-precision applications where sample integrity and gentle handling are prioritized over simplicity.

**Table 6:** Overview of Dielectrophoretic, and Acoustic Wave-based Plasma Separation Methods and Performance Parameters.

Reference	Sample Volume	Extraction Efficiency	Yield	Blood Sample	Extraction Time	Hematocrit Level	Final Purpose
I-Dielectrophoretic methods							
[68]	10 μL	99.98% ± 0.02%	2.2 μL	Whole Blood	4 min	15–65%	Plasma separation
[69]	5 μL	90%	10–100 nL	Whole Blood	4 min	NA	Determination of blood lithium-ion concentration
II-Acoustic Wave-based methods							
[71]	30 μL/min	~100%	55.6%	Whole Blood	~250 ms	40%	Microparticle separation
[72]	115 µL/min	>99.99%	23 µL/min	Whole Blood	Cont. System	NA	Blood sampling and plasma separation
[73]	NA	>90%	NA	Whole Blood	NA	NA	WBC/RBC/platelet separation
[74]	3.5 mL	58.42% for plateletes, 99.96% for RBCs	NA	Diluted with PBS	50 μL/min	NA	Blood cell separation
[75]	5 mL	NA	NA	Diluted blood with PBS	200 s	~50%	Measuring viscosity and aggregation of blood sample

NA—Not applicable.

#### 3.2.5. Magnetic Separation-Based Techniques

Magnetic separation-based techniques for plasma isolation exploit the selective labeling or manipulation of blood components with magnetic materials to achieve field-driven separation. In these systems, erythrocytes are typically functionalized/coated with magnetic nanoparticles or magnetic beads conjugated to antibodies targeting cell-surface markers, enabling their rapid removal under an externally applied magnetic field while cell-free plasma is directed toward downstream analysis. Microfluidic platforms further optimize this strategy by incorporating spatial magnetic gradients, magnetophoretic pathways, and precisely regulated flow profiles, collectively improving capture performance while reducing unintended cell retention.

**Figure 12:**
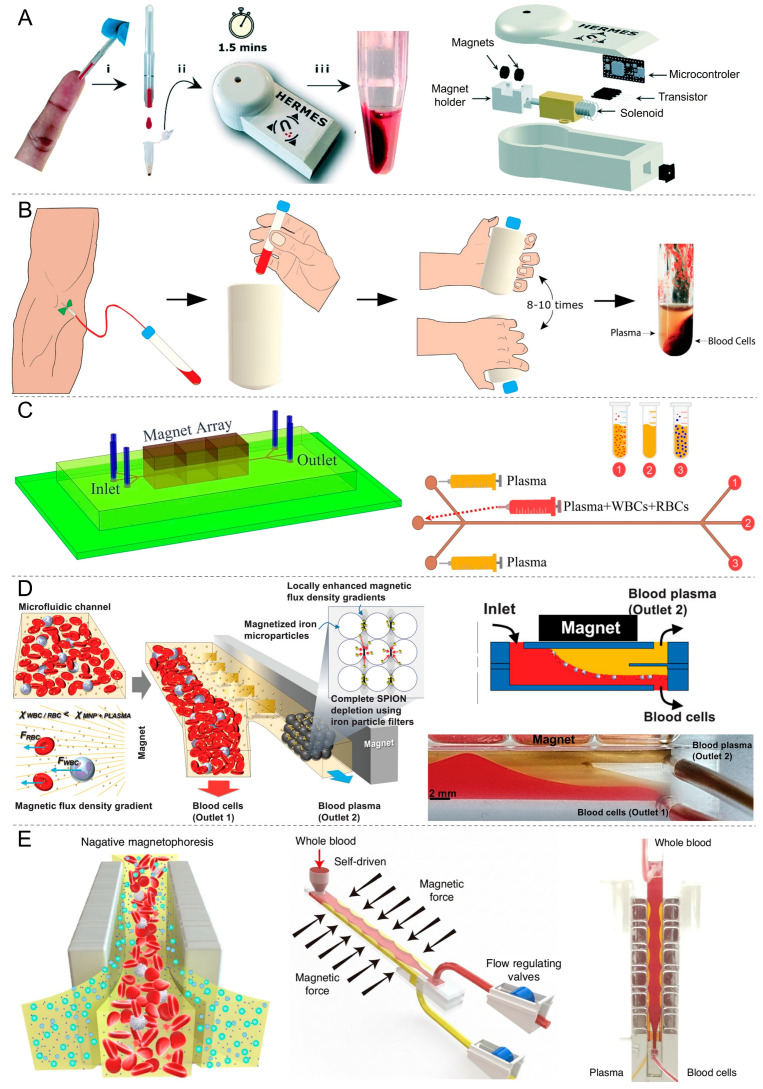
Microfluidic platforms based on magnetic separation use magnetic fields and magnetic particles for label-free plasma separation from whole blood. (**A**) illustrates the H.E.R.M.E.S workflow, in which whole blood loaded into a bead-coated tube undergoes magnetically actuated RBCs aggregation and capture on the tube wall [76]. (**B**) shows the H.E.R.M.E.S sleeve, which enables rapid magnetic bead-based separation of plasma from up to 1 mL of whole blood through simple manual mixing and subsequent magnetic capture [77]. (**C**) shows a microfluidic device that uses plasma cladding streams and a Halbach array of permanent magnets to apply differential magnetophoretic forces for continuous separation of RBCs and WBCs [78]. (**D**) describes a microfluidic plasma-separation system that uses SPION-enhanced diamagnetic repulsion and a Halbach magnet array to drive blood cells away from the flow path, enabling continuous collection of cell-free plasma near to the magnet [79]. (**E**) shows a plasma-separation device that uses ferrofluid-enhanced negative magnetophoretic forces generated by an alternating double Halbach array to drive blood cells toward the center of the channel while enabling purified plasma collection through a static separation channel [80].

A magnetic-based plasma separation device, High Efficiency Rapid Magnetic Erythrocyte Separator (H.E.R.M.E.S), separates plasma from 40 µL whole blood samples without dilution and consistently yields ~17 µL of uncontaminated plasma (>90% of available serum) with >99.9% purity (Figure 12A) [76]. The total extraction time is approximately 108 s, driven by magnetic bead-based erythrocyte capture enhanced through aggregation. The system operates effectively across varying hematocrit levels, including artificially elevated samples up to 70–90%, still producing high-purity plasma. The device demonstrates high extraction efficiency at a cost of less than $2 per test and delivers robust performance independent of hematocrit level.

Updated version of the H.E.R.M.E.S, H.E.R.M.E.S sleeve, was introduced that processes up to 1 mL of whole blood without dilution and relies on magnetic bead-based erythrocyte capture enhanced by aggregation to achieve high-performance plasma separation (Figure 12B) [77]. The device with sleeve delivers ~76% plasma yield while maintaining ~99.9% purity. Separation requires only 45 s of gentle inversion (8–10 times), followed by magnetic settling, making the total extraction time well under two minutes. The device operates effectively at a normalized hematocrit of ~50%, and the aggregation mechanism is expected to remain functional even in high-hematocrit or high-viscosity samples. The sleeve provides a resource-independent plasma separation method suitable for field or mobile testing.

A microfluidic magnetophoretic system separates plasma from whole blood by introducing the blood into a low-flow central stream (0.5–2.5 µL/h) while plasma cladding streams guide and focus the sample for efficient magnetophoretic separation (Figure 12C) [78]. The device achieves high cell separation efficiency for specific combinations of core/cladding flow rates and magnet distances, but it does not generate high plasma volume as an output. The study demonstrates that magnetophoretic force balance and optimized flow-rate ratios govern the system’s ability to achieve complete cell separation.

A diamagnetic repulsion-based microfluidic device was introduced that operates on the principle of diamagnetic repulsion, in which superparamagnetic nanoparticles (SPIONs) added to whole blood create a paramagnetic plasma environment that forces inherently diamagnetic blood cells to migrate away from the plasma stream under a magnetic field gradient (Figure 12D) [79]. This enables processing of undiluted whole blood at a physiological hematocrit of 45%, handling volumes up to 4 mL and flow rates reaching 100 µL/min without requiring dilution. Using this mechanism, it was reported that the system achieves complete (100%) removal of blood cells while maintaining a high plasma yield of 83.3 ± 1.64%, and the recovered plasma maintains its biochemical integrity. Blood cells are displaced at velocities near 199 µm/s, allowing rapid and continuous plasma extraction within the channel.

Another device operates on the principle of negative magnetophoresis, in which whole blood mixed with a biocompatible ferrofluid is exposed to an enhanced double Halbach magnetic array that repels non-magnetic blood cells and allows plasma to migrate toward the channel walls (Figure 12E) [80]. In whole rat blood, the platform yields 40 μL plasma from 100 μL input within 8 min, achieving a plasma recovery rate of 72.7% and a blood cell removal efficiency of 99.9%. In human blood, larger cell size enables much faster operation, allowing 3 mL of plasma to be separated in 1 min.

Magnetic separation-based approaches (Table 7) provide a controlled way to remove blood cells by applying external magnetic fields, either through magnetically labeled cells or inherent magnetophoretic behavior. This technique enables continuous, on-chip operation and allows cell removal to occur without relying on physical filters or large external hardware.

## 4. Conclusions and Future Directions

Microfluidic plasma separation has emerged as one of the most significant enablers for the development of point-of-care diagnostic platforms, offering rapid, miniaturized, and straightforward alternatives to traditional centrifugation. Each technique demonstrates unique strengths and limitations, yet collectively they illustrate the versatility of microfluidic engineering in addressing long-standing challenges related to blood handling, including the need for reduced sample volume, accelerated extraction times, and compatibility with downstream molecular assays.

Passive methods have demonstrated strong potential for low-resource settings due to their simplicity, ease of fabrication, and cost-effectiveness. However, issues such as membrane clogging, dependency on hematocrit levels, and limited yield continue to constrain their robustness in clinical workflows. Active techniques, by contrast, provide higher precision in separating plasma from cellular components and enable real-time control of flow dynamics, making them suitable for integration with high-performance diagnostic modules. Nevertheless, they often require external energy input or sophisticated instrumentation, raising concerns about scalability and accessibility in decentralized healthcare environments. This trade-off between operational simplicity and separation performance remains at the core of current microfluidic plasma separation research.

In addition to the engineering aspects, biological variability introduces another dimension of complexity. Patient-to-patient variations in hematocrit, viscosity, and coagulation tendencies directly impact device performance, underscoring the importance of designing platforms that are resilient to these physiological differences. Equally important is the integration of plasma separation modules with downstream diagnostic workflows, whether for nucleic acid amplification, protein biomarker detection, or metabolite analysis. True clinical utility will be realized only when plasma extraction, target isolation, and readout are seamlessly coupled in a unified, low-cost device that requires minimal operator intervention.

Looking ahead, several promising directions can shape the future of this field. The incorporation of novel separation materials, including nanostructured membranes, bioinspired coatings, and stimuli-responsive polymers, offers new opportunities to improve throughput and minimize fouling. Advances in 3D printing and scalable manufacturing techniques, such as injection molding and roll-to-roll processing, are likely to accelerate the translation of microfluidic plasma separation devices into commercial products. Furthermore, artificial intelligence and machine learning may be applied to optimize device design, predict separation outcomes across heterogeneous blood samples, and guide automated quality control. Integration of digital health interfaces, such as smartphone-based imaging and cloud-linked data analytics, may further expand the accessibility and impact of plasma-based diagnostics in both developed and resource-limited regions.

Future advances in microfluidic plasma separation are likely to be driven more by system-level integration strategies than by isolated technological improvements. A critical direction is the development of hematocrit-agnostic platforms, where device performance is intrinsically stable across physiological variability rather than tuned for narrow operating windows. Separation will shift from maximizing purity to achieving analyte-specific preparation, focusing on selectively isolating biomarker-rich fractions like extracellular vesicles, circulating tumor cells or mitigating analyte interference from specific hemolysis products. The field is also likely to move toward architecture-level standardization, in which reusable separation motifs (e.g., sedimentation-first, membrane-refinement, field-assisted polishing) are adapted across platforms instead of repeatedly reinvented.

Despite these advances, significant challenges remain. Regulatory approval and standardization frameworks must be established to validate the reproducibility, reliability, and safety of microfluidic plasma separation technologies. Ethical and practical considerations, such as equitable access, affordability, and deployment in low-infrastructure healthcare systems, also require careful attention. Ultimately, the success of microfluidic plasma separation will depend not only on technical innovations but also on interdisciplinary collaboration across engineering, clinical medicine, regulatory science, and industry.

In summary, plasma separation on microfluidic platforms represents a rapidly maturing technology that holds transformative potential for clinical diagnostics, personalized medicine, and global health. By addressing current challenges and leveraging emerging innovations, this field is poised to move beyond proof-of-concept demonstrations and deliver standardized, scalable, and clinically validated solutions. The path forward demands a deliberate balance between simplicity, performance, and integration, ensuring that microfluidic plasma separation fulfills its promise as a cornerstone of next-generation diagnostic technologies.

## Figures and Tables

**Table 1:** Overview of filtration-based plasma separation methods and performance parameters.

Reference	Sample Volume	Extraction Efficiency	Yield	Blood Sample	Extraction Time	Hematocrit Level	Final Purpose
[16]	50 µL	NA	56.88%	Whole blood	87 s	NA	Capillary blood plasma separation
[17]	20 μL	45%	3 μL	Whole blood	20 min	NA	Detection of cardiac protein markers
[18]	~40 µL	97%	NA	Diluted with PBS-EDTA	12–15 min	NA	C-reactive protein testing
[19]	NA	99.8%	5–30 μL	Whole blood	5 min	48%	Plasma separation
[20]	100–200 μL	99.8%	5–30 μL	Whole blood	20 min	48%	Accurate plasma separation
[21]	200 μL	NA	~50 μL	Whole blood	6–10 min	NA	Detection of multiple chronic disease biomarkers
[22]	100 μL	96%	NA	Whole blood	3 min	NA	HIV viral load quantification
[23]	2.3 mL	NA	~130 μL	Whole blood	8 min	NA	Plasma separation
[24]	1.8 mL	95.5 ± 3.5% to 81.5 ± 12.1%	275 ± 33.5 µL	Whole blood	<7 min	NA	Plasma separation for HIV detection
[25]	~115–120 µL	53.8%	~65.6 ± 3.9 µL	Whole blood	10 min	20–50%	Immunochromatographic assay for tetanus antibodies
[26]	400 µL	~100%	~131.8 ± 3.4 µL	Whole blood	5 min	65%	Immunocapture plasma separation for clinical assays

NA—Not applicable.

**Table 3:** Overview of superhydrophobic membrane-based, and paper-based plasma separation methods and performance parameters.

Reference	Sample Volume	Extraction Efficiency	Yield	Blood Sample	Extraction Time	Hematocrit Level	Final Purpose
I-Superhydrophobic membrane-based methods							
[38]	200 µL	>84.5 ± 25.8%	65 ± 21.5 µL	Whole blood	<10 min	NA	Plasma separation
[39]	10–100 µL	99.99%	~80%	Whole blood	20–80 s	~45%	Plasma separation
II-Paper-based methods							
[40]	10–40 μL	99.99%	~3.3 μL (60.1%)	Whole Blood	5 min	~45%	Plasma separation on Chinese Xuan papers
[41]	80 μL	NA	NA	Whole Blood	2–4 min	27–55%	Non-enzymatic electrochemical detection of ascorbic acid
[42]	300 μL	98%	50 μL	Whole Blood	220 s	45%	Detection of protein Biomarkers
[43]	NA	NA	NA	Whole Blood	1 min	NA	NA detection
[44]	30 μL	NA	~10 μL	Whole Blood	11 min	30–50%	Multi-target biochemical analysis
[45]	73.3 μL	99.91%	>80%	Whole Blood	75 s	NA	Detection of CRP and PAB
[46]	7 μL	72%	90–110%	Whole Blood	NA	39.98 ± 4.3%	Plasma separation on filter paper using the anti-H agglutinating antibody

NA—Not applicable.

**Table 4:** Overview of active pump-assisted plasma separation methods and performance parameters.

Reference	Sample Volume	Extraction Efficiency	Yield	Blood Sample	Extraction Time	Hematocrit Level	Final Purpose
[49]	5 μL	98.5%	NA	Diluted with PBS	<1 min	NA	Plasma separation
[50]	0.6 mL	NA	100 µL	Diluted with PBS	>5 min	NA	Analysis of glucose, cholesterol from whole blood
[51]	1 mL	98% for RBC and 96% WBC	250–300 µL	Whole Blood	40–65 min	45–55%	Platelet-rich plasma separation
[52]	3 mL	~99.99%	NA	Diluted with PBS	30 min	~25%	MicroRNA and extracellular vesicle analysis
[53]	5 mL	NA	NA	Diluted with physiological salt solution (PSS)	20 min	NA	Surface modification of PDMS microfluidics for plasma separation
[54]	180 μL	97%	NA	Diluted blood with PBS	3 min	NA	Plasma separation with pneumatic peristaltic micropump
[55]	NA	99.70% including PLTs	NA	Diluted blood	NA	0.1–3%	Plasma separation
[56]	5 μL	NA	1 μL	Whole Blood	2 min	Adult donor blood	Determination of glucose concentration in blood
[57]	400 μL	NA	100 μL	Whole Blood	7 min	<50%	C-reactive protein (CRP) detection

NA—Not applicable.

**Table 5:** Overview of centrifugation-based plasma separation methods and performance parameters.

Reference	Sample Volume	Extraction Efficiency	Yield	Blood Sample	Extraction Time	Hematocrit Level	Final Purpose
[59]	15 µL	~99.99%	~93%	Whole Blood	250 s	10–60%	Plasma separation
[60]	10 µL	99% for RBC and 93% WBC	NA	Whole Blood	10 min	~45%	Hematocrit measurement, white blood cell counting and plasma separation
[61]	3 mL	99%	1.3 mL	Whole Blood	300 s	45%	Cell-free fetal DNA extraction
[62]	70 μL	NA	37.7 μL	Whole Blood	8 min	45%	Plasma separation
[63]	2 mL	>99%	40–70%	Whole Blood	5 min	<20–50%	Effect of siphon valve on plasma separation of whole blood with different HCT levels.
[64]	50 μL	99%	NA	Whole Blood	50 s	48%, 24%, 12%	Early detection of pepsinogen
[65]	20 μL	99.99%	NA	Whole Blood	7 min	NA	Plasma separation and bio-sensing
[66]	3 mL	99.992%	90%	Whole Blood	300 s	45%	Plasma separation with using separator gel on disk

NA—Not applicable.

**Table 7:** Overview of magnetic-based plasma separation methods and performance parameters.

Reference	Sample Volume	Extraction Efficiency	Yield	Blood Sample	Extraction Time	Hematocrit Level	Final Purpose
[76]	40 µL	99.9%	17.2 ± 1.96 µL	Whole blood	108 ± 21 s	70–90%	Plasma separation
[77]	1 mL	99.9%	76.7 ± 11.5%	Whole blood	2 min	50%	Plasma separation
[78]	5 µL/h	~100%	NA	Whole blood	NA	NA	Plasma separation by Halbach array
[79]	4 mL	NA	83.3 ± 1.64%	Whole blood	100 µL/min	NA	Plasma separation for biomarker analysis
[80]	100 μL	99.9%	40 µL	Whole blood	~8 min	NA	Plasma separation for biochemical analysis

NA—Not applicable.

## Data Availability

Not applicable.
